# Isolation and Analysis of Matched Osteoarthritic Cartilage Progenitor Cells and Bone Marrow Mesenchymal Stem Cells

**DOI:** 10.7759/cureus.80844

**Published:** 2025-03-19

**Authors:** Adam Esa, Naveed Ahmed, Mohamed F Elsheikh, Hesham Ahmed, Rayan A Cherif, Charles Archer

**Affiliations:** 1 Trauma and Orthopedics, Cardiff University, Cardiff, GBR; 2 Trauma and Orthopedics, Prince Charles Hospital, Merthyr Tydfil, GBR; 3 Trauma and Orthopedics, Wrexham Maelor Hospital, Wrexham, GBR; 4 College of Medicine, Cardiff University, Cardiff, GBR; 5 Regenerative Medicine, Swansea University, Swansea, GBR

**Keywords:** articular cartilage, chondroprogenitor cells, osteoarthritis, regenerative medicine, stem cells

## Abstract

Introduction: Osteoarthritis (OA) is a chronic degenerative disorder that impacts synovial joints, leading to the degradation of articular cartilage and alterations in bone structure. As the most prevalent type of polyarthritis, its occurrence is increasing, particularly in Western countries. Current treatment options for OA involve various pharmacological therapies and prosthetic devices, which come with numerous limitations. Consequently, there is a growing interest among both patients and health care professionals in biological therapies, particularly the use of stem and progenitor cells for cartilage regeneration.

Methods: We extract articular cartilage progenitor cells (CPCs) and bone marrow mesenchymal stem cells (MSCs) from the femoral side of the knee joint of OA patients undergoing total knee arthroplasty. To isolate CPCs, digested full-depth chondrocytes from the femoral condyle undergo a fibronectin adhesion assay, while we separate bone marrow MSCs using the Ficoll™ density gradient centrifugation method. We expand both cell types in culture and measure their growth kinetics over 80 days. Additionally, we evaluate proliferation potential and senescence through bromodeoxyuridine incorporation and the senescence-associated β-galactosidase assay, respectively. Further, we analyze the expression of specific MSC markers in articular CPCs and bone marrow MSCs using flow cytometry.

Results: We successfully isolated CPCs and bone marrow MSCs from matched osteoarthritic donors. The isolated CPCs and MSCs exhibit similar morphology and proliferation ability. Moreover, both cell types show positive expression for MSC markers CD-90, CD-105, and CD-166, while expressing low or no levels of CD-34 (a marker for hematopoietic stem cells) and exhibiting tri-lineage differentiation potential.

Conclusion: We successfully isolate CPCs and bone marrow MSCs from the knee joints of osteoarthritic donors. Our findings indicate that both cell types demonstrate comparable morphology and growth kinetics, concurrently marking for classical MSC markers and exhibiting differentiation potential. These results are promising for the field of regenerative medicine. In this study, we outline the isolation of a rare group of matching mesenchymal stem/progenitor cells collected from the articular cartilage and bone marrow of patients undergoing total knee arthroplasty. This discovery lays the groundwork for comparative analyses, in that these cell types are primary candidates for cartilage-based regenerative therapies.

## Introduction

Joint arthroplasty is the primary treatment for end-stage osteoarthritis (OA), involving surgical intervention. Although it is effective for advanced OA, this procedure has notable limitations. These include the surgery’s inherent complexities, the intensive post-operative care required, the eventual wear of prosthetic implants, and the risk of surgical site infections. Moreover, the increase in life expectancy, coupled with a rise in sports-related injuries among younger people, presents challenges for many patients considering arthroplasty due to the technical difficulties associated with repeated surgical revisions [1-3]. As a result, many patients are excluded from this treatment avenue, which leads to prolonged discomfort and emphasizes the critical need for alternative therapeutic options. The societal impact of OA is further underscored by its economic burden, particularly through lost productive workdays, affecting both individuals and the overall economy [1,4,5].

Articular cartilage forms through the differentiation of mesenchymal stem cells (MSCs) during chondrogenesis. It was traditionally believed that articular cartilage lacked regenerative capabilities due to the perceived absence of resident stem or progenitor cells. Nonetheless, this view has been challenged by the discovery of chondroprogenitor cells, which offer promising avenues for enhancing cartilage regeneration techniques [6]. Researchers are investigating various cell sources for their potential in cell-based tissue engineering of articular cartilage, such as articular cartilage progenitor cells (CPCs) and MSCs. Research conducted by McCarthy et al. revealed that CPCs demonstrate better chondrogenic characteristics than bone marrow-derived mesenchymal stem cells (BM-MSCs) in equine models [7]. Although this finding is promising, it is still uncertain if similar outcomes can be achieved with human-derived cells. Furthermore, the aim of cell-based therapies for OA is to halt or reverse the progression of the disease through the transplantation of cartilage. Nevertheless, uncertainties persist concerning the viability of mesenchymal progenitor cells derived from OA patients as a potential source for cartilage regeneration [1,6,8-10].

Our first aim in this study was to characterize and compare CPC populations obtained through the fibronectin cell adhesion assay from full-depth chondrocytes of articular cartilage in patients with OA and those with normal joint tissue.

The second aim was to assess the MSC properties of CPCs from both normal donors and OA patients and compare these properties with matched BM-MSCs from the same OA patients.

## Materials and methods

Isolation of human cartilage chondrocytes and CPCS

Articular cartilage was harvested from the knee joints of two groups: normal donors (n=5) and patients undergoing total knee arthroplasty (n=6). The dissected cartilage underwent enzymatic digestion to release chondrocytes, followed by the fibronectin adhesion assay to isolate articular CPCs. For each donor, full-depth chondrocytes were seeded onto a fibronectin-coated six-well tissue culture plate [6]. Initial cell adhesion was assessed, and colony-forming efficiency (CFE) was calculated based on the data obtained from the initial seeding [1,6]. 

Colony-forming potential of CPC isolated from OA and normal human articular cartilage

Ten days post-plating, we analyzed the normal (n=6 wells per donor) and OA (n=6 wells per donor) donor CPC using microscopy and calculated the CFE as follows: CFE=(Number of colonies at day 10>32 cells)/(Number of the initial cells)*100.

Immunofluorescence labeling

Fixed cells were blocked with goat serum (50 μg/mL) for 30 minutes at room temperature. After removing the blocking serum, primary antibodies diluted in PBS containing 1% BSA were applied, and the sections were incubated overnight at 4°C. The sections were then washed twice with PBS-Tween (5 minutes per wash) and incubated with the appropriate secondary antibody for 1 hour at room temperature. The secondary antibodies used included goat anti-mouse IgG conjugated to Alexa Fluor 488, 594, or 647 for mouse IgG primary antibodies; goat anti-rabbit IgG conjugated to Alexa Fluor 633 for polyclonal rabbit primary antibodies; and goat anti-mouse IgM conjugated to Alexa Fluor 594 for mouse IgM primary antibodies. All secondary antibodies were diluted to a final concentration of 10 μg/mL. After processing, the sections were mounted in Vectashield (Vector Labs, USA) containing either 4,6-diamidino-2-phenylindole (DAPI) or propidium iodide to counterstain cell nuclei. Imaging was performed using a Leica DM2500 or DM6000 upright confocal microscope (Leica, Germany).

Peroxidase labeling

Fixed cells were treated with 0.3% (v/v) hydrogen peroxide in water for 30 minutes at room temperature to inhibit endogenous peroxidase activity. The sections were rinsed three times with PBS-Tween (5 minutes per wash) and blocked with the R.T.U. Vectastain Universal Quick Kit blocking serum (horse serum) for 30 minutes. Excess serum was removed, and primary antibodies, diluted in PBS-Tween, were applied directly to the sections, followed by incubation at 4°C overnight.

BrdU assay

Cells were suspended in basal medium and seeded at a density of 10⁴ cells per well in 12-well cell culture plates. The plates were incubated for 24 hours at 37°C in a humidified atmosphere with 5% CO₂. Triplicate wells were prepared for each cell line. After incubation, the medium was replaced with basal medium containing 10 µM bromodeoxyuridine (BrdU), and the cells were incubated for an additional 24 hours. Following this, the cells were washed twice with PBS and fixed in ice-cold 70% (v/v) ethanol for 30 minutes at room temperature. After fixation, the cells were washed again with PBS and stored at 4°C for subsequent analysis.

To detect incorporated BrdU, subsequent immunodetection was performed using a specific G3G4 anti-BrdU mouse monoclonal antibody (DSHB, USA) at 3 µg/mL for 1 hour at room temperature. Prior to antibody incubation, cell layers were treated with 1 M HCl to denature the DNA. After completing the protocol, the plates were dried, and glycerol was added to each well as a mounting medium. Cells were visualized using a Nikon Eclipse TS100 light microscope, and images were captured from each well (n = 3) using a Nikon E4500 camera with a 20x objective. The total number of cells and BrdU-positive cells were counted, and the percentage of stained cells was calculated by dividing the number of positively stained cells by the total cell count using ImageJ software (National Institutes of Health, Bethesda, Maryland).

Senescence-associated β-galactosidase staining

Cells in the basal medium were seeded at 10⁴ cells per well in 12-well cell culture plates and incubated for 24 hours at 37°C in a humidified atmosphere with 5% CO₂. Triplicate wells were prepared for each cell line. After incubation, the cells were washed twice with PBS-Tween and fixed in a solution of 2% formaldehyde and 0.2% glutaraldehyde in PBS for 5 minutes at room temperature. Following fixation, the cells were washed with PBS and incubated in β-galactosidase staining solution (40 mM citric acid/phosphate buffer, 1 mg/mL X-Gal, 150 mM NaCl, 2 mM MgCl₂, 5 mM K₄(Fe(CN)₆), 5 mM K₃(Fe(CN)₆), pH 6) for 16 hours at 37°C. After staining, the cells were washed twice with PBS and twice with methanol, and the plates were air-dried.

The plates were examined using an inverted Nikon Eclipse TS100 light microscope, and images were captured from each well (n=3) using a Nikon E4500 camera with a 20x objective. Senescent cells were identified by the presence of blue β-galactosidase deposits in the cytoplasm. The total number of cells and stained cells were counted, and the percentage of stained cells was calculated by dividing the number of positively stained cells by the total cell count using ImageJ software.

Flow cytometry

Confluent cell layers were detached using 5 mL of TryPLE solution (Life Technologies, UK), washed in DMEM-PS, and counted using a hemocytometer. Routinely, 1 × 10⁶ cells were resuspended in 1 mL of ice-cold PBS containing 0.1% (w/v) BSA. Aliquots of 10⁵ cells (100 µL) were transferred to 1.5 mL sterile microcentrifuge tubes and pelleted at 400 × g for 5 minutes. The supernatant was removed, and the cell pellets were resuspended in 100 µL of PBS/BSA containing 10 µg/mL of the appropriate primary conjugated antibody (see Table 2). The cells were incubated in the dark at 4°C for 30 minutes, with an appropriate mouse IgG used as a negative control. After incubation, the cells were centrifuged, washed twice with 1 mL of PBS/BSA to remove unbound antibodies, and resuspended in 500 µL of PBS/BSA. Each cell suspension was transferred to a 12 mm fluorescence-activated cell sorting (FACS) tube (BD Biosciences, UK) for analysis using a FACS Canto flow cytometer. Data were processed using FACS Diva software (BD Biosciences).

Cells were analyzed based on size and granularity using forward and side scatter, respectively, to differentiate viable cells. Data were displayed as density plots, and IgG isotype controls were overlaid as histograms to assess specificity. The data were analyzed using Flowing Software (v2.5, University of Turku, Finland). Antibody expression levels were calculated as the ratio of the geometric mean fluorescence intensity (MFI) of positively stained cells to that of the isotype control.

**Table 1:** Conjugated primary antibodies used for flow cytometry PerCP-eFluor® 710, peridinin-Chlorophyll Protein Complex eFluor® 710; APC, allophycocyanin; PE, phycoerythrin; FITC, fluorescein Isothiocyanate

Antibody name	Clone name	Conjugated type	Source	Species
Anti-CD90 (Thy-1)	5E10	PerCP-eFluor® 710	ebioscience	Mouse (IgG1, κ)
Anti-CD105	SN6	APC	ebioscience	Mouse (IgG1, κ)
Anti-CD166	3A6	PE	ebioscience	Mouse (IgG1, κ)
Anti-CD-34	QBEND	FITC	Abcam	Mouse (IgG1)
Isotype Control	P3.6.2.8.1	APC	ebioscience	Mouse IgG1 K
Isotype Control	P3.6.2.8.1	PerCP-eFluor® 710	ebioscience	Mouse IgG1 K
Isotype Control	P3.6.2.8.1	PE	ebioscience	Mouse IgG1 K
Isotype Control	P3.6.2.8.1	FITC	ebioscience	Mouse IgG1 K

RNA extraction from cells

Total RNA was routinely isolated from cell cultures using Trizol® (Invitrogen, UK) at a concentration of 1 mL per 0.5-1 × 10^6^ cells, following the manufacturer’s protocol. Trizol was added to cells, which were lifted by repetitive pipetting and transferred to a 1.5 mL RNase-free microcentrifuge tube. Then, 200 μL of molecular-grade chloroform was added, and the samples were mixed by inversion before standing for 15 minutes at room temperature. The samples were centrifuged for 15 minutes at 13,000 × g at 4°C. The top aqueous phase, containing the RNA, was carefully removed without disturbing the DNA interface layer and transferred to a fresh 1.5 mL microcentrifuge tube. An equal volume of 70% ethanol was added to the aqueous phase, and the sample was carefully mixed by inversion.

RNA was isolated using the RNeasy Mini Kit according to the manufacturer’s protocol. Briefly, the sample was transferred into an RNeasy spin column placed in a 2 mL collection tube and centrifuged at 8,000 × g for 30 seconds. 350 μL of RW1 buffer was added to the spin column, and it was centrifuged at 8,000 × g for 30 seconds. To eliminate genomic DNA contamination, an on-column DNase digestion step was carried out by adding 80 μL of DNase solution (10 μL of DNase I + 70 μL of RDD buffer) onto the center of the RNeasy spin column membrane and leaving it for 15 minutes at room temperature. Subsequently, 350 μL of RW1 buffer was added to the spin column, and it was centrifuged at 8,000 × g for 30 seconds. RPE buffer (500 μL) was then added to the RNeasy column and centrifuged at 8,000 × g for 30 seconds. Another 500 μL of RPE buffer was added to the RNeasy column, and it was centrifuged for another 30 seconds at 8,000 × g. The RNeasy spin column was then centrifuged at 8,000 × g for 2 minutes to remove any remaining buffer. The spin column was transferred to a new 0.5 mL RNase-free microcentrifuge tube, and the RNA was eluted with 30 μL of RNase-free water using a final spin at 8,000 × g for 1 minute. RNA was quantified using a Nanodrop 2000c spectrophotometer by measuring the 260/280 nm absorbance ratio, and the RNA was stored at -80°C until required.

Analysis of RNA using the Agilent 2100 Bioanalyzer

The integrity of RNA (5 μL per sample) was assessed using the Agilent 2100 Bioanalyzer (Dr. Claudia Consoli, Central Biotechnology Services, Cardiff University). The Agilent Bioanalyzer is an electrophoresis-based system for detecting RNA and proteins. It measures the integrity and identifies any degraded material that might affect the purity of isolated total RNA. Twelve samples were assessed for RNA integrity, and RNA was visualized on a gel, with sharp bands representing the ribosomal 28S and 18S (the top and bottom bands were clearly demarcated, respectively). No evidence of RNA degradation was observed.

Reverse transcription of isolated RNA

cDNA was synthesized by reverse transcription of total RNA (500 ng) using SuperScript™ III Reverse Transcriptase, following the manufacturer’s protocol (Invitrogen, UK). RNA (500 ng) was added to 1 μL of random hexamers (250 ng final concentration) and 1 μL of dNTPs (500 μM), with the final volume made up to 10 μL using molecular biology-grade water. The samples were incubated at 65°C for 5 minutes in a thermocycler, followed by incubation on ice for at least 1 minute. Next, 0.5 μL of SuperScript™ III Reverse Transcriptase (100 U final concentration), 4 μL of 5X first-strand buffer, 2 μL of DTT (100 mM), 1 μL of recombinant RNase inhibitor (125 U), and 2.5 μL of molecular grade water were added. The samples were placed in a thermocycler programmed for 10 minutes at 25°C, followed by 50 minutes at 42°C. Finally, the reaction was terminated by heating the samples to 70°C for 15 minutes. The cDNA reaction (20 μL) was immediately used or stored at -20°C until required.

Quantitative real-time PCR

PCR primers for each gene of interest (Table 2) were designed using NCBI software to span exon-exon boundaries. The selected primers were then run through the NCBI BLAST database to ensure no homology with other genes. Primers were purchased from Sigma (UK) and optimized prior to use in quantitative real-time PCR (qPCR) using the GoTaq Flexi DNA Polymerase kit (Promega, UK) according to the manufacturer’s protocol. PCR reaction products were analyzed on 1% (w/v) agarose gels containing Safeview (10 μL per 40 mL) by electrophoresis in 0.5X TBE buffer at 75V for 40-50 minutes and visualized under UV light. A molecular weight marker (100-1000 bp) was used to identify product sizes.

**Table 2:** PCR primer sequence All primers are shown in the 5’-3’ with annealing temperature and size. PCR, polymerase chain reaction

Gene ID	Sequence	Annealing temperature and size	Accession number
PPAR-γ F PPAR-γ-R	AAAGAAGCCAACACTAAACC TGGTCATTTCGTTAAAGGC	61ºC 78bp	|NM_138711.3
ACAN-F ACAN-R	TGAGTTTCCTGGTGTGAG AGACCTCACCCTCCATC	61ºC 100bp	NM_001135.3
Runx-2 F Runx-2 R	AAGCTTGATGACTCTAAACC TCTGTAATCTGACTCTGTCC	62ºC 164bp	NM_001278478.1
LPL-F LPL-R	ACACAGAGGTAGATATTGGAG CTTTTTCTGAGTCTCTCCTG	60ºC 105bp	NM_000237.2
Osteonectin-F Osteonectin-R	AGTATGTGTAACAGGAGGAC AATGTTGCTAGTGTGATTGG	60ºC 143bp	NM_003118.3
Col2α1-F Col2α1-R	GAAGAGTGGAGACTACTGG CAGATGTGTTTCTTCTCCTTG	60ºC 165bp	NM_001844.4
GAPDH-F GAPDH-R	ACAGTTGCCATGTAGACC TTTTTGGTTGAGCACAGG	60ºC 95bp	NM_001256799.2

Quantification of mRNA levels for genes of interest was performed using qPCR with SYBR® Green KicqStart™ q-PCR ReadyMix™ (Sigma, UK). A 20 μL reaction volume was set up in a 96-well plate (Applied Biosystems) as follows: 7 μL RNAase-free H₂O, 1 μL each of forward and reverse primers (0.3 μM), 10 μL KicqStart™ TaqReadyMix™, and 2 μL cDNA (from 500 ng RNA). qPCR reactions were carried out using an Mx3000P q-PCR system (Stratagene, UK) with the following amplification conditions: 95°C for 10 minutes (one cycle), 95°C for 30 sec, annealing temperature (see Table 2) for 30 sec, 72°C for 30 sec for 40 cycles, followed by a final extension at 72°C for 5 minutes, and then 4°C. Relative quantification was analyzed using the 2-ΔΔCT method. Briefly, normal and full-depth chondrocytes and CPCs were used as control groups to measure relative changes in the target gene expression in osteoarthritic full-depth chondrocytes and CPC samples. The relative change in gene expression was presented as a fold change, normalized to GAPDH.

Multipotency of derived CPC lines

Monolayer cultures of polyclonal CPC lines from normal donors (n=3), OA donors (n=3), and matched BM-MSCs (n=3) were trypsinized, washed, and counted. The cells were then processed to assess their differentiation potential toward chondrogenic, osteogenic, and adipogenic lineages.

Chondrogenic differentiation

Cell pellets were re-suspended in DMEM + Glutamax with 100 mg/mL gentamicin, 50 μg/mL ascorbic acid, 1% HEPES buffer, and supplemented with 1% insulin, transferrin, selenium (ITS), 0.1 μM dexamethasone, and 10 ng/mL TGF-β2 (chondrogenic medium) at 1.2 × 10⁶ cells/mL. The cells were seeded into Corning® 96-well ultra-low attachment spheroid microplates at 100,000 cells per well and incubated at 37°C in a humidified atmosphere containing 5% CO₂. CPCs formed aggregates within the first 24 hours, and the medium was changed three times weekly for three weeks. Afterward, pellets were collected for mRNA isolation and analysis of chondrogenic differentiation gene expression using RT-PCR or fixed in 70% (v/v) ethanol for Toluidine blue staining and Safranin-O staining [6].

Osteogenic differentiation

Cell pellets were re-suspended at 0.2 × 10⁶ cells/mL in medium consisting of DMEM containing 100 mg/mL Gentamicin, 10% (v/v) fetal bovine serum (FBS), 50 μM ascorbic acid 2-phosphate, 10 mM β-glycerol phosphate, 10 nM dexamethasone, and 1% (v/v) HEPES buffer (osteogenic medium), and plated into 24-well tissue culture dishes at 50,000 cells per well [6]. Cells were cultured for 21 days at 37°C in a humidified atmosphere containing 5% CO₂, and the medium was changed three times a week. After 21 days of culture in an osteogenic medium, cell layers were washed briefly with PBS and either fixed using a 4% (w/v) paraformaldehyde fixative solution (4°C for 20 minutes) or mRNA was extracted for analysis of osteogenic differentiation gene expression using RT-PCR.

Fixed cell layers were washed with PBS and then stained using Alizarin Red Stain solution (2 mg/mL alizarin red in 0.5 M acetic acid, pH 4.2, adjusted with 1% (v/v) ammonium hydroxide) for 20 minutes at room temperature to detect the presence of calcium deposits. Cells were washed five times with PBS to remove any unbound stain and were visualized and photographed using a Nikon TE-DH100w camera attached to a Nikon Eclipse TE300 microscope (Nikon, UK).

Adipogenic differentiation

Cell pellets were re-suspended at 0.2 × 10⁶ cells/mL in an adipogenic differentiation medium consisting of DMEM containing 100 mg/mL Gentamicin, 10% (v/v) FBS, 0.5 mM isobutylmethylxanthine (IBMX), 100 μM indomethacin, 1 μM dexamethasone, and 10 μg/mL insulin. Cells (50,000 cells per well) were maintained in an adipogenic differentiation medium for one week, and the medium was changed three times per week. After the experimental duration, cells were washed with PBS and either fixed with 4% (v/v) paraformaldehyde for 15 minutes at room temperature or mRNA was extracted for analysis of adipogenic differentiation gene expression using RT-PCR [6].

Freshly fixed cells were stained with Oil Red O (0.3% (v/v) Oil Red O in 60% isopropanol, made from a freshly prepared stock solution of 0.5% (w/v) Oil Red O in 100% isopropanol) to detect lipid droplets in the differentiated cells, as per the published protocol [6]. The stain was applied to the plates for 1 hour at room temperature before being washed thoroughly with distilled water. Staining was visualized and photographed using a Nikon TE-DH100w camera attached to a Nikon Eclipse TE300 microscope (Nikon, UK).

Statistical analysis

All quantitative data are presented as mean ± standard error of the mean (SEM) and were analyzed using the "R" statistical software (version 2.15.0) (R Foundation for Statistical Computing, Vienna, Austria). For comparisons between the two groups, a Student’s t-test was employed. The normality of the data distribution was assessed using the Shapiro-Wilk test, with log transformation applied when necessary. The homogeneity of variance was confirmed using the Fligner test (F-test). Once homogeneity was established, differences in variances between groups were evaluated using a one-way analysis of variance (ANOVA), followed by Tukey’s post-hoc test for multiple comparisons. A P-value of <0.05 was considered statistically significant. All experiments were repeated at least three times unless otherwise specified. Additionally, correlation analysis was performed using the built-in Pearson’s correlation function in the software to assess relationships between parametric variables.

## Results

Initial adhesion of isolated chondrocytes from OA and normal human articular cartilage

**Figure 1:**
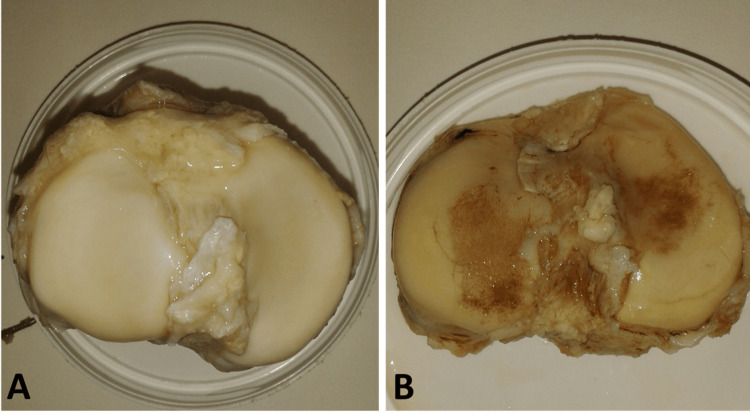
Tibial plateau isolated from normal and osteoarthritic donors The tibial plateau from a normal donor exhibits a smooth, intact articular cartilage surface with no visible abnormalities (A). In contrast, the osteoarthritic tibial plateau (B) displays characteristic features of OA, including cartilage erosion and exposure of the subchondral bone. OA, osteoarthritis

**Figure 2:**
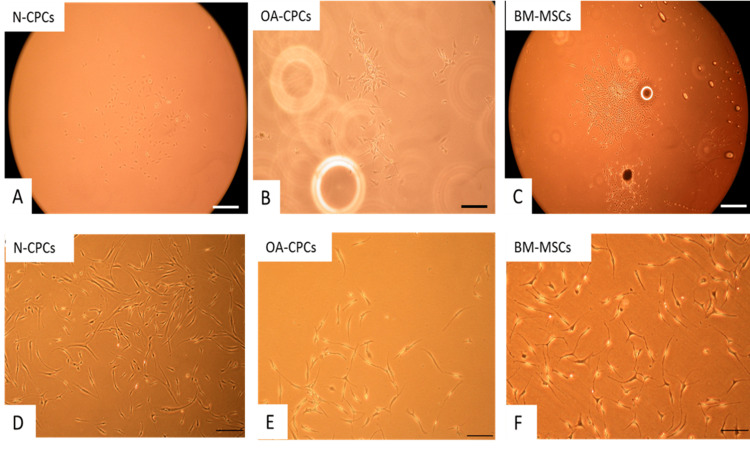
Phase contrast images of CPCs and MSC (A) Colony-forming chondroprogenitor cells (CFU) from N-CPC. (B) OA-CPC. (C) Patient-matched BM-MSCs. (D) Isolated N-CPC after culture expansion. (E) OA-CPC after culture expansion. (F) Culture-expanded bone marrow MSCs. Scale bar: A-C = 100 µm. Scale bar: D-F = 200 µm. N-CPC, normal cartilage progenitor cell; BM-MSCs, bone marrow-derived mesenchymal stem cells; MSCs, mesenchymal stem cells; OA, osteoarthritis; CPC, cartilage progenitor cell

**Figure 3:**
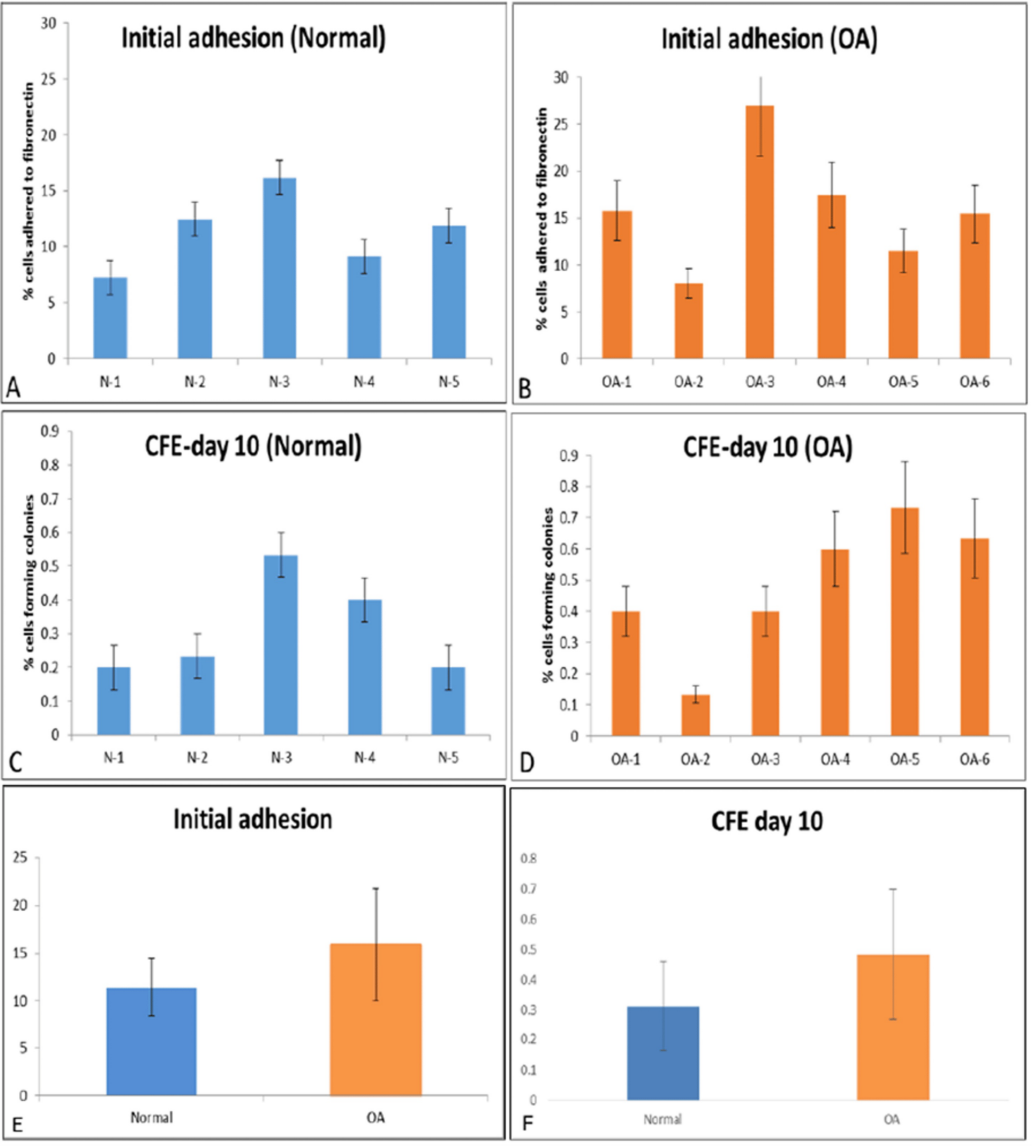
Initial adhesion to fibronectin and CFE A and B display the initial percentage of chondrocytes adhering to fibronectin 24 hours after plating, with data from normal donors (n=5) and osteoarthritic donors (n=6), respectively. C and D illustrate the CFE at day 10 for CPCs isolated from normal (n=5) and OA donors (n=6). CPCs, cartilage progenitor cells; CFE, colony-forming efficiency; OA, osteoarthritis

We extracted articular cartilage from the proximal tibia of six patients with OA (mean age 66 years, range 53-80) and five normal donors (mean age 43 years, range 29-55) (Figure 1). Chondrocytes were obtained from the digested cartilage, and the initial adhesion of the cells was evaluated after seeding onto fibronectin-coated tissue culture wells (n = 6 wells per donor) through microscopy seven days post-plating (Figure 2). The percentage of cells adhering to fibronectin from the five normal donors varied between 7.2% and 16%, whereas those from the six OA donors ranged from 8% to 29%. Data from both donor groups exhibited a normal distribution, and there was no statistically significant difference in the percentage of cells initially adhering to fibronectin between normal and OA cartilage (Figure 3, P > 0.05). These adherent cells were labeled as CPC.

CFE in CPCs derived from OA donor cartilage varied from 0.1% to 0.73%, whereas CFE from CPCs isolated from normal donor cartilage ranged from 0.06% to 0.53% (see Figure 3). Both groups exhibited a normal distribution, and the percentage of CFE showed no statistically significant difference between normal and OA CPCs (Figure 3, p = 0.34). By day 10 of culture, we either isolated colonies of CPCs in each of the 6 × 35 mm wells for expansion as monoclonal cell lines or further cultured them as polyclonal cell lines. Data were collected from six wells per donor, with 1,000 chondrocytes per 35 mm well placed on fibronectin-coated plates. Statistical analysis demonstrated a normal distribution for both donor groups, and no significant difference was found in initial cell adhesion (p > 0.05) or CFE percentages between normal and OA CPCs (Figure 3, p = 0.34).

Comparison of cell morphology of polyclonal CPC lines (from cartilage of normal and OA donors) and matched BM-MSCs (from OA donors)

Within four days after plating, polyclonal CPCs from both normal and OA sources formed small colonies of a few cells. By day 10, numerous colonies containing more than 32 cells were visible. OA-derived BM-MSCs adhered to tissue culture plastic within 24 hours, with colonies noticeable between four and 10 days after seeding. During culture expansion, both cell types exhibited a spindle-like morphology, with no clear morphological differences found among polyclonal normal CPC (Figure 2), OA-CPC (Figure 2), and OA-BM-MSCs (Figure 2). 

Analysis of proliferating cells in CPC lines originating from cartilage of normal and OA donors and matched BM-MSCs from OA donors

**Figure 4:**
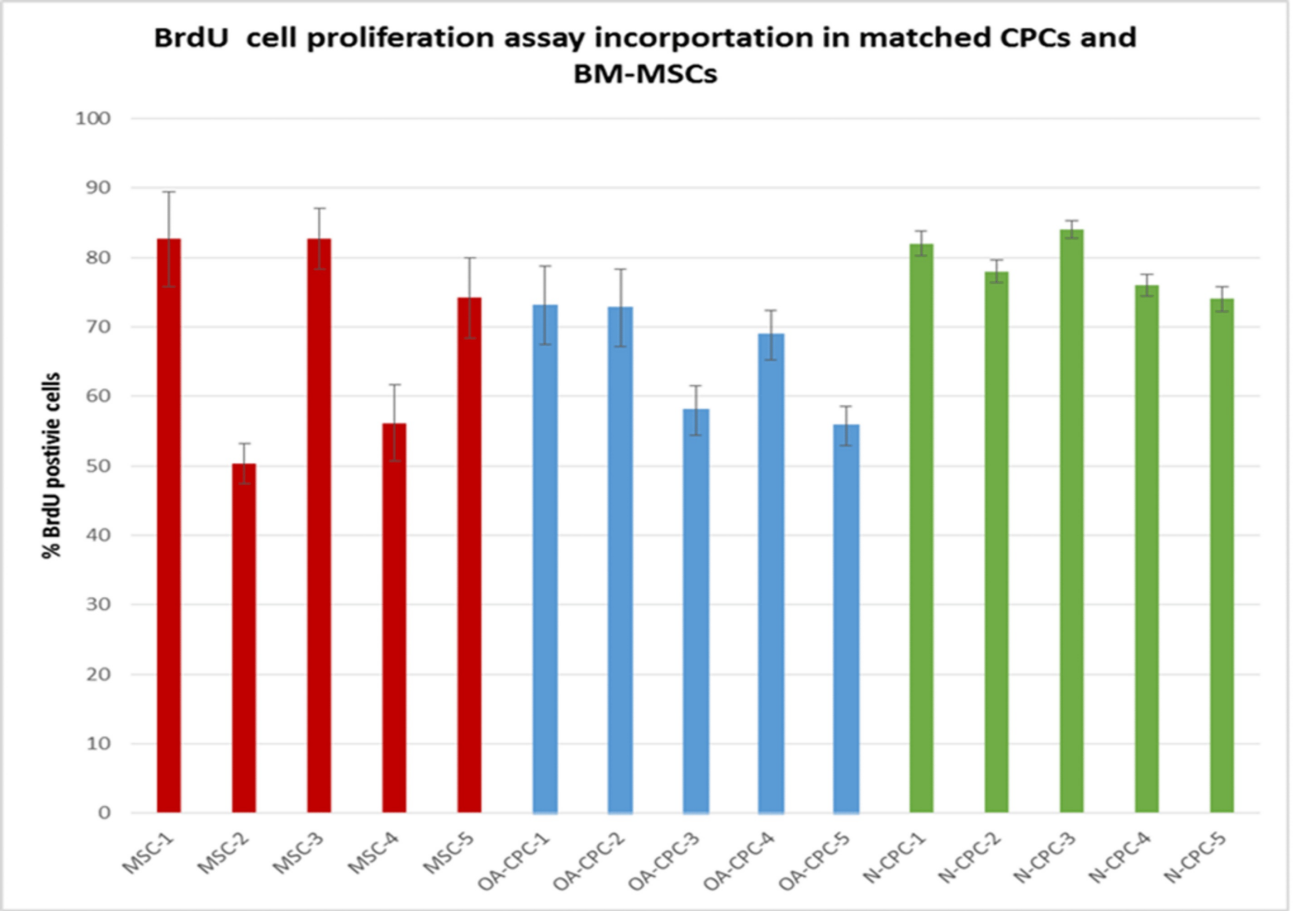
BrdU (5-bromo-2-deoxyuridine) cell incorporation The percentage of positively stained cells was calculated for each cell line (n = 3), with data presented as mean ± SEM. Blue bars represent OA-CPCs, red bars correspond to matched MSCs, and green bars indicate N-CPCs. N-CPC, normal cartilage progenitor cell; BM-MSCs, bone marrow-derived mesenchymal stem cells; OA, osteoarthritis; CPC, cartilage progenitor cell; BrdU, bromodeoxyuridine

We measured the proliferation of polyclonal OA-CPC lines, along with matched OA-BM-MSCs and polyclonal normal cartilage progenitor cell (N-CPC) lines, at a population doubling of 25 (±2.5) using the BrdU assay (Figure 4). Three repeats were performed for each cell line. Variations were observed in the percentage of BrdU-positive cells from each donor (Figure 4), with the mean percentage of BrdU-positive cells for the OA-CPC and matched OA-BM-MSCs donors calculated as 69.18% (± 5.07%) and 65.69% (± 4.23%), respectively, while 78.8% (± 1) of cells from N-CPC donors labeled positive for BrdU. All data sets showed a normal distribution, and no significant differences in cell proliferation were observed between the selected cell lines (p > 0.05).

Analysis of senescent cells in CPC lines originating from cartilage of normal and OA donors and matched BM-MSCs from OA donors

**Figure 5:**
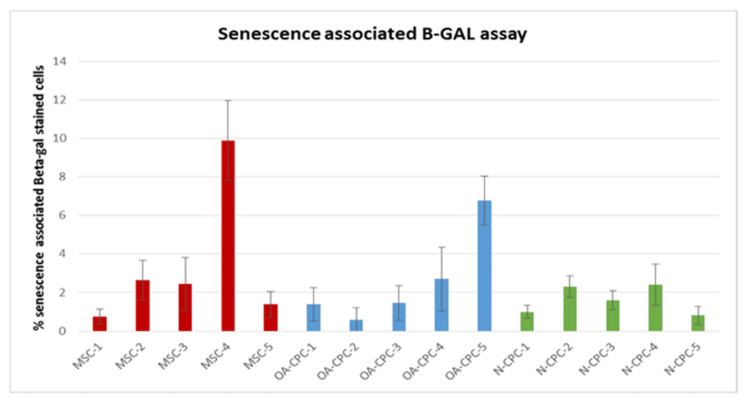
Percentage of senescent cells in normal CPCs, OA-CPCs, and BM-MSCs BM-MSCs, bone marrow-derived mesenchymal stem cells; OA, osteoarthritis; CPC, cartilage progenitor cell

**Figure 6:**
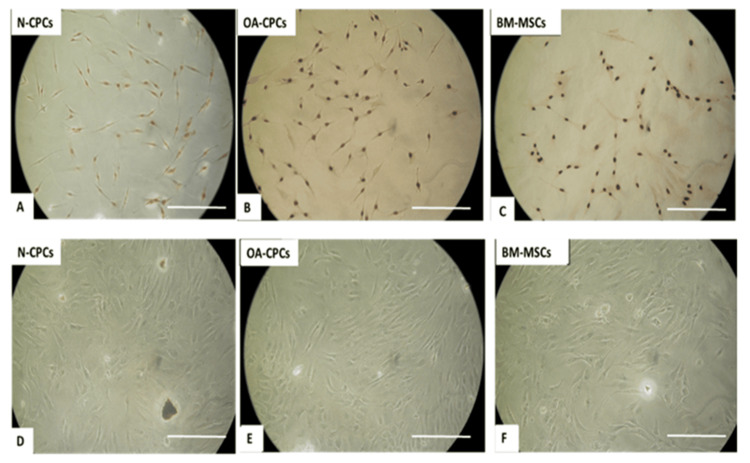
BrdU labeling and senescence-associated β-galactosidase assay of CPCs and BM-MSCs Representative images of CPCs isolated from normal (A, D) and osteoarthritic (B, E) cartilage, as well as BM-MSCs (C, F), stained for BrdU (A-C) and β-galactosidase (D-F). Scale bar = 200 µm. BM-MSCs, bone marrow-derived mesenchymal stem cells; OA, osteoarthritis; CPC, cartilage progenitor cell; N-CPC, normal cartilage progenitor cell; BrdU, bromodeoxyuridine

We assessed the percentage of senescent cells in cultures of polyclonal OA-CPC lines, along with matched bone marrow-derived OA-BM-MSCs and polyclonal N-CPC lines, at a population doubling of 25 (±2.5) using the senescence-associated β-galactosidase assay (Figure 5 and Figure 6). Each cell line underwent three trials. The senescent cell percentages for the OA-CPC-derived line and its corresponding OA-BM-MSCs were found to be 0.59%-6.7% and 0.7%-9.8%, respectively, whereas the N-CPC-derived lines recorded a range of 0.8%-2.4%. All data sets exhibited a normal distribution, with mean senescent cell percentages of 3.41% (± 1.11%) for OA-BM-MSCs, 2.58% (± 1.05%) for OA-CPC, and 1.6% (± 0.58%) for N-CPC. Data showed no significant differences in cell senescence among the selected cell lines (p > 0.05).

**Figure 7:**
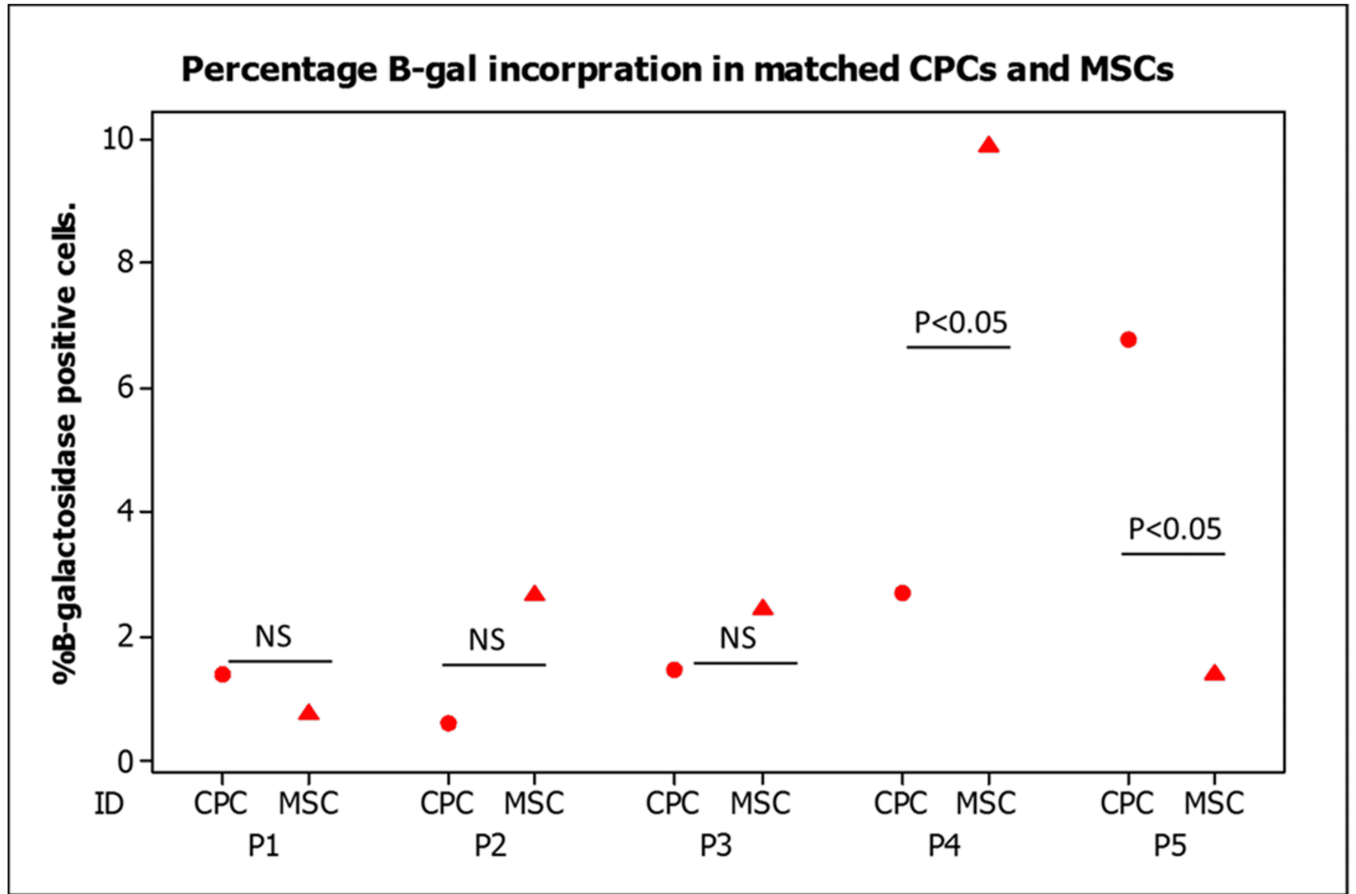
Percentage of B-galactosidase incorporation in matched CPC and MSCs The percentage of β-galactosidase incorporation was measured in matched CPC lines derived from osteoarthritic cartilage (circles) and BM-MSCs (triangles) from five donors. Each cell line was analyzed in triplicate. Data are presented as mean values, and statistical differences were assessed using a Student’s t-test (P < 0.05). BM-MSCs, bone marrow-derived mesenchymal stem cells; CPC, cartilage progenitor cell; NS, not significant

We assessed the senescence activity of polyclonal CPCs alongside their matched BM-MSCs at a population doubling of 25 (± 2.5) using the corresponding β-Gal staining assay. Analysis of interpatient β-Gal incorporation revealed differences in the percentage of positive senescent cells across each donor (Figure 7). The data showed no significant differences in the percentage of β-Gal incorporation between CPCs and MSCs in three patients (p > 0.05). However, in the remaining two patients with statistically significant interpatient variations, one patient’s MSCs demonstrated higher BrdU incorporation (p < 0.05), whereas the other patient exhibited greater β-Gal incorporation in the CPC line compared to the matched MSCs (p < 0.05).

Expression of MSC surface markers in CPC isolated from normal and OA donor cartilage with matched BM-MSCs from the OA donors

**Figure 8:**
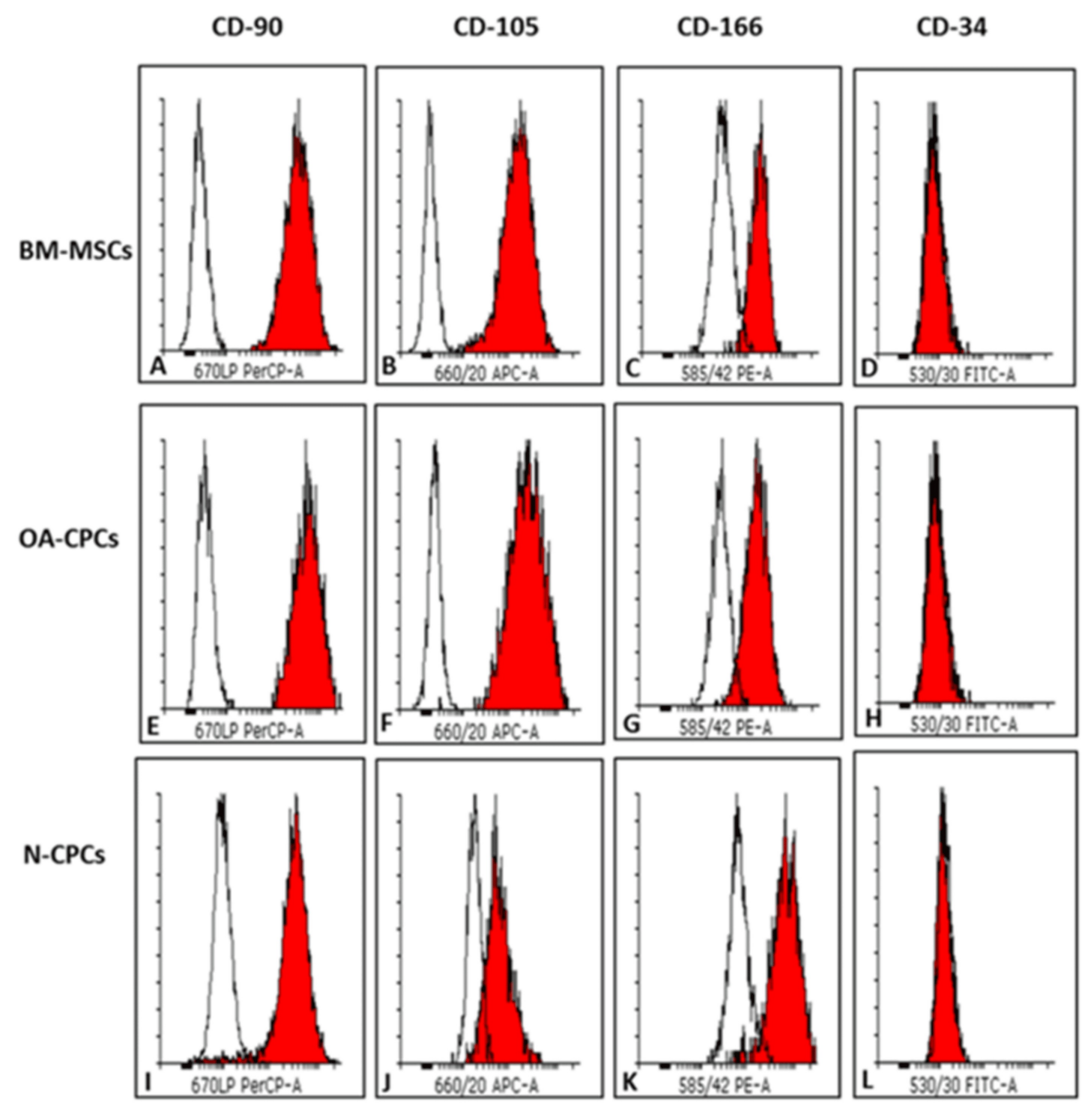
FACS analysis of MSC surface markers FACS analysis was conducted to assess the expression of CD-90, CD-105, CD-166, and CD-34 in CPC lines derived from both normal and osteoarthritic cartilage, as well as in OA-BM-MSCs. Representative histograms are shown for an OA-BM-MSC line (A-D), an OA-CPC line (E-H), and an N-CPC line (I-L). CD-34, a hematopoietic stem cell marker used as a negative control for MSCs, is displayed in D, H, and L for each respective cell line. The red-filled histogram represents positively labeled cells, while the unfilled histogram corresponds to the matched isotype control. FACS, fluorescence-activated cell sorting; OA-BM-MSCs, OA, osteoarthritis-matched bone marrow mesenchymal stem cells; BM-MSCs, bone marrow-derived mesenchymal stem cells; N-CPC, normal cartilage progenitor cell; OA, osteoarthritis; CPC, cartilage progenitor cell

We assessed the expression levels of MSC-associated surface biomarkers (CD-90, CD-105, and CD-166) in N-CPC, OA-CPC, and corresponding OA-BM-MSC lines through flow cytometry (FACS). CD-34, recognized as a negative marker for MSCs, served as a control. Cell lines were selected at equivalent population doublings, with one cell line analyzed from each donor. Figure 8 presents representative histograms from a single donor’s OA-BM-MSCs, N-CPCs, and OA-CPCs.

All donors showed strong expression of CD-90, CD-105, and CD-166, with average expression exceeding 95% in gated live cells, highlighting a uniform population. In contrast, polyclonal N-CPCs demonstrated an average CD-166 expression of 92.6% in gated cells positive for this stem cell marker. The negative marker CD-34 showed mean expression levels of 0.02% in N-CPCs, 0.25% in OA-CPCs, and 0.53% in OA-BM-MSCs (p < 0.05). Although CD-34 expression was slightly elevated in OA-BM-MSCs compared to OA-CPCs and N-CPCs, it remained within the acceptable limits for defining a pure MSC population as specified by the International Society for Cellular Therapy (ISCT) [1,9-11].

Analysis of multi-lineage differentiation of CPC and BM-MSCs 

We initiated chondrogenic differentiation using 3D pellet cultures involving polyclonal N-CPCs, polyclonal OA-CPCs, and matched OA-BM-MSCs from three different donors. These cultures were maintained for 21 days before termination. For each donor and cell line, 3D pellet cultures were created in 24 wells of a 96-well plate, with 12 wells designated for RNA analysis and the other 12 prepared for histochemical staining.

**Figure 9:**
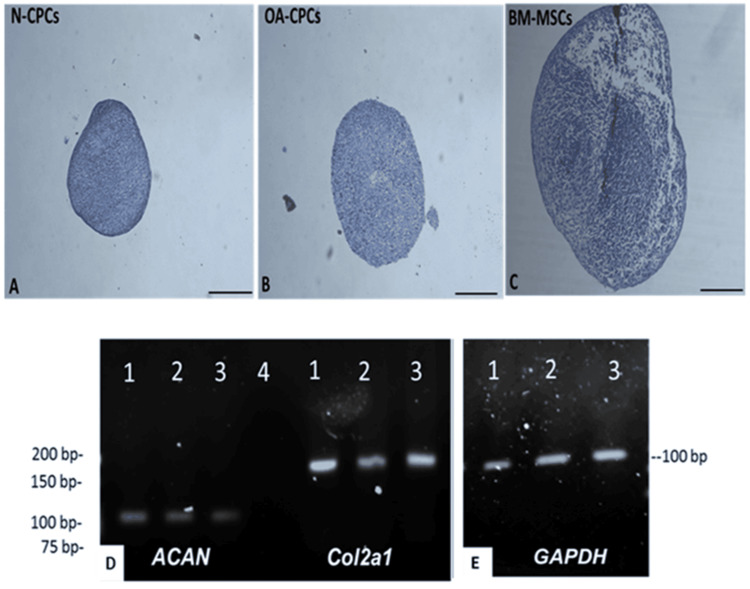
Chondrogenic differentiation of isolated CPC and MSCs A-C: Representative Toluidine blue-stained images of 3D pellet cultures from N-CPC (A), OA-CPC (B), and matched OA-BM-MSC (C) lines after 21 days of differentiation. D-E: Representative PCR analysis showing mRNA expression of aggrecan (ACAN, 100 bp), type II collagen (COL2A1, 165 bp), and GAPDH (positive control, 95 bp). Toluidine blue staining confirmed the presence of a glycosaminoglycan-rich matrix in all tested cell lines. Lane 1: N-CPC, Lane 2: OA-CPC, Lane 3: OA-BM-MSCs, Lane 4: NTC. Scale bars = 50 μm. NTC, no-template control; BM-MSCs, bone marrow-derived mesenchymal stem cells; N-CPC, normal cartilage progenitor cell; OA, osteoarthritis; CPC, cartilage progenitor cell

After 21 days of culture, Toluidine blue staining indicated glycosaminoglycan synthesis in all tested cell lines (Figure 9). Visually, the pellets produced by N-CPCs and OA-CPCs were slightly smaller and denser than those from OA-BM-MSCs. RT-PCR analysis of the extracted mRNA revealed the expression of aggrecan (ACAN) and collagen II (COL2A1) following chondrogenic differentiation, with detectable levels of these gene transcripts present in all cell lines (Figure 9).

**Figure 10:**
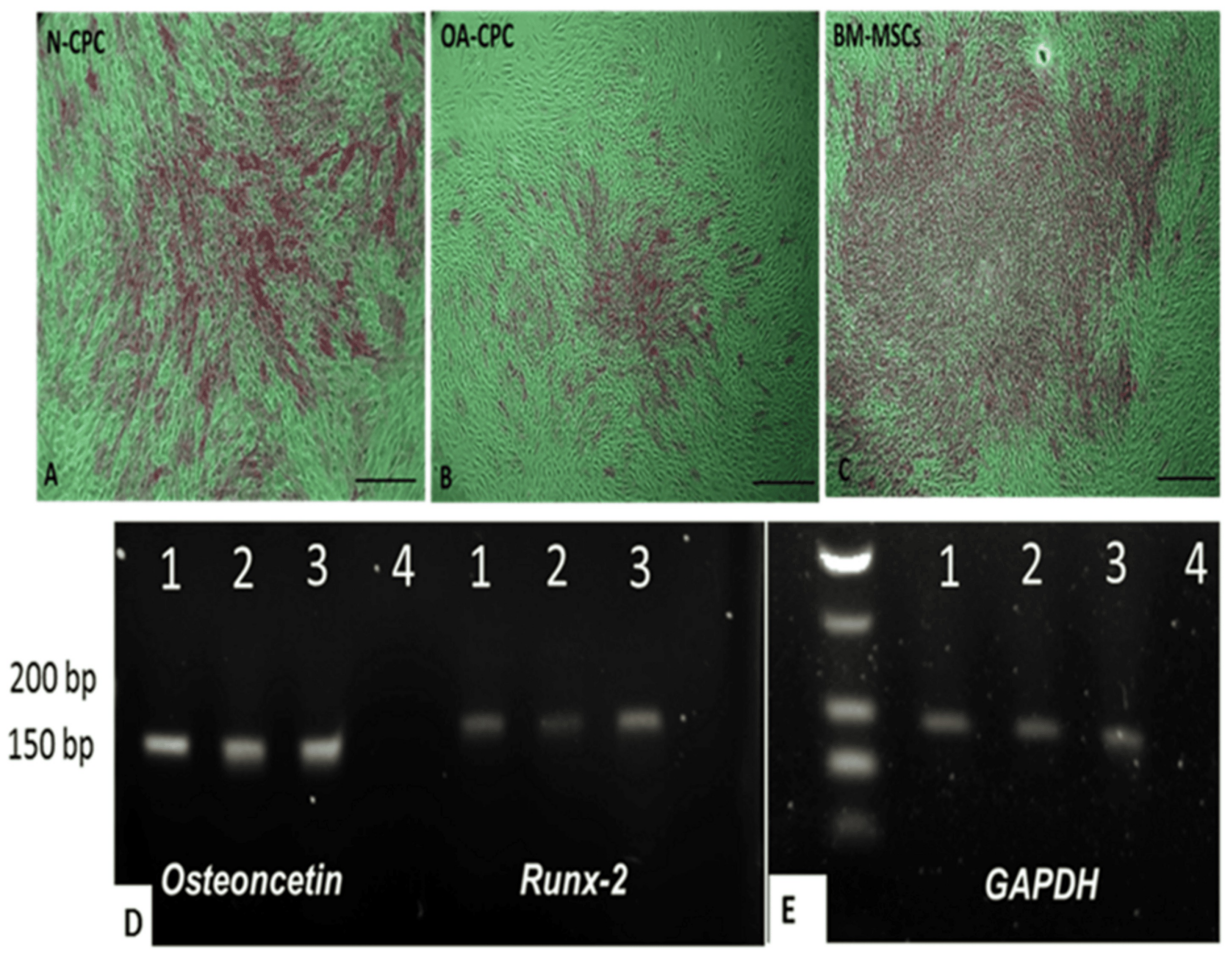
Osteogenic differentiation of isolated CPC and MSCs A-C: Representative images of Alizarin Red staining in monolayer cultures of N-CPC, OA-CPC, and OA-BM-MSC lines after 14 days in an osteogenic medium. Alizarin Red staining was used to assess calcium deposition, a key marker of osteogenic differentiation. D-E: Representative PCR analysis showing mRNA expression of Runx-2 (164 bp), Osteonectin (143 bp), and GAPDH (control, 95 bp) in N-CPC, OA-CPC, and matched OA-BM-MSCs. All cell lines exhibited positive staining for Alizarin Red, indicating mineral deposition. Lane 1: N-CPC, Lane 2: OA-CPC, Lane 3: BM-MSCs, Lane 4: NTC. Scale bars = 50 μm. NTC, no-template control; BM-MSCs, bone marrow-derived mesenchymal stem cells; N-CPC, normal cartilage progenitor cell; OA, osteoarthritis; CPC, cartilage progenitor cell

We induced osteogenic differentiation in monolayer cultures using polyclonal N-CPCs, polyclonal OA-CPCs, and matched OA-BM-MSCs from three donors. The cultures were maintained for 14 days, after which differentiation was evaluated through Alizarin Red staining to identify mineral deposition and RT-PCR analysis of osteogenic markers Runx-2 and Osteonectin. Alizarin Red staining revealed similar staining intensity for N-CPCs and OA-BM-MSCs, suggesting mineralized deposition. Conversely, OA-CPC cultures also displayed calcium deposition, but some areas in the wells showed insufficient staining (Figure 10). RT-PCR analysis confirmed the expression of osteogenic differentiation markers, with all three cell lines, N-CPCs, OA-CPCs, and OA-BM-MSCs, expressing mRNA for Osteonectin and Runx-2, reinforcing the mineralization patterns observed through Alizarin Red staining (Figure 10).

**Figure 11:**
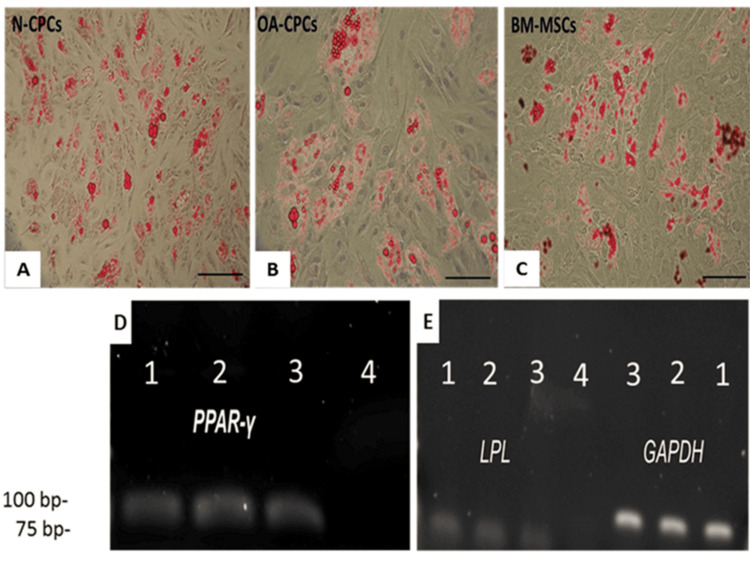
Adipogenic differentiation of CPC and MSCs A-C: Representative images of Oil Red O staining in monolayer cultures of N-CPC, OA-CPC, and OA-BM-MSC lines after one week in adipogenic medium. Lipid droplet accumulation is visible as red staining in all cell lines. D-E: Representative RT-PCR analysis of adipogenic differentiation markers, including PPAR-γ (78 bp) (D) and LPL (105 bp) (E), confirming adipogenic induction in monolayer cultures. GAPDH (95 bp) served as the housekeeping gene (E). Lane 1: N-CPC, Lane 2: OA-CPC, Lane 3: OA-BM-MSCs, Lane 4: NTC. Scale bars = 50 μm. PPAR-γ, peroxisome proliferator-activated receptor-γ; LPL, lipoprotein lipase; NTC, no-template control; BM-MSCs, bone marrow-derived mesenchymal stem cells; N-CPC, normal cartilage progenitor cell; OA, osteoarthritis; CPC, cartilage progenitor cell

We initiated adipogenic differentiation in monolayer cultures of N-CPCs, OA-CPCs, and corresponding OA-BM-MSCs from three donors. Lipid staining indicated adipogenic differentiation in CPC lines derived from both normal and OA cartilage, as well as in their matched BM-MSCs, corroborating the differentiation capacity of these cell lines (Figure 11). RT-PCR analysis revealed the expression of adipogenic markers, including peroxisome proliferator-activated receptor gamma (PPAR-γ) (Figure 11) and lipoprotein lipase (LPL) (Figure 11).

## Discussion

In this study we aimed to achieve two main objectives: first, to compare CPCs sourced from normal and OA cartilage; and second, to characterize and analyze CPCs from both normal and OA cartilage alongside BM-MSCs obtained from OA donors. BM-MSCs are well-researched for their multipotent capabilities and their potential role in tissue regeneration. They have been utilized in conjunction with autologous chondrocytes in clinical studies, including the ASCOT trial, whose instigators focus on repairing focal cartilage lesions in younger patients [1,9,12].

Researchers effectively isolated CPCs from chondrocytes obtained from both macroscopically healthy and OA cartilage through fibronectin adhesion assays [1,6,9,13]. Additionally, matched BM-MSCs were extracted from OA patients using Ficoll™ density gradient centrifugation. To reduce variability, investigators specifically obtained CPCs and BM-MSCs from the femoral condyle of the knee joint. The isolation and characterization of CPCs derived from OA are particularly important because they could provide an allogeneic cell source for cartilage repair.

Initial studies on morphology, fibronectin adhesion, and CFE showed that normal and OA-derived CPCs shared similar morphological characteristics. Statistically, there were no significant differences in initial adhesion to fibronectin or CFE between the two groups. Dowthwaite et al. (2004) found a mean CPC adhesion rate of 9% in immature bovine cartilage [14], which is consistent with our results of 11.3% for normal CPCs and 15.8% for OA-CPCs in human cartilage. Likewise, Williams et al. (2010) and Nelson et al. (2014) reported OA-CPC adhesion rates ranging from 10.7% to 15.4% [6,9].

The CFE of polyclonal OA-CPCs (0.1%-0.73%) was marginally higher compared to normal CPCs (0.06%-0.53%), although this variation was not statistically significant (p > 0.05). These findings align with previous research indicating CFE values below 1% when using fibronectin adhesion assays [6,9,14,15]. Notably, Grogan et al. (2009) identified similar proportions of mesenchymal progenitor cells in both OA and healthy cartilage through FACS sorting of Hoechst 33342-labeled cells [16]. Conversely, other investigators have reported larger proportions (10%-15%) of stem/progenitor cells isolated via FACS with MSC markers [9,15-18]. This difference may suggest the presence of transient amplifying cells, which show elevated levels of α5β1 integrin but do not proliferate during clonal expansion [13].

All cell lines showed sustained proliferation for over 80 days in culture, consistent with existing literature [1,6,15]. BrdU assays demonstrated significant proliferative potential in normal CPCs (78.8% ± 1%), OA-CPCs (69.18% ± 5.07%), and corresponding OA-BM-MSCs (65.69% ± 4.23%), suggesting comparable proliferative abilities among these three cell types [1,19].

We evaluated cell senescence using the senescence-associated β-galactosidase assay [20]. The proportion of senescent cells was 3.41% (± 1.11%) for OA-BM-MSCs, 2.58% (± 1.05%) for OA-CPCs, and 1.6% (± 0.58%) for normal CPCs, showing no significant differences among the groups. Although Yu et al. (2011) reported higher senescence-associated β-galactosidase activity with increasing age [21], our results revealed no correlation between donor age (mean 43 years for normal donors and 66 years for OA donors) and rates of senescence. The observed lower senescence in normal CPCs compared to OA-CPCs and BM-MSCs may be due to limitations in sample size, which warrants further investigation.

In 2006, the ISCT established minimal criteria for identifying MSCs, which include the expression of CD105, CD73, and CD90, and the absence of CD45, CD34, CD14, CD11b, CD19, and HLA-DR [1,10,11]. Despite these established guidelines, researchers have observed differences in marker expression and differentiation potential among MSCs derived from various tissues [16,22-24]. In this study, all cell types adhered to tissue culture plastic and exhibited a spindle-like morphology characteristic of MSCs [25]. Importantly, both normal and OA-derived CPCs, as well as matched OA-BM-MSCs, expressed the mesenchymal markers CD90, CD105, and CD166. Although the expression levels of CD90 and CD166 were similar among the cell types, CD105 showed slightly reduced expression in normal CPCs (95%) compared to OA-derived cells (99%). Additionally, all cell types demonstrated minimal expression of CD34, indicating no endothelial cell contamination [1].

All cell types were able to differentiate into chondrocytes, osteoblasts, and adipocytes. Chondrogenic differentiation was confirmed through toluidine blue staining, which indicated glycosaminoglycan production, along with mRNA expression of specific lineage markers. These results align with previous studies highlighting the multi-lineage potential of CPCs [1,6]. A unique aspect of this research is the characterization of patient-matched CPCs and BM-MSCs, which minimizes source bias and enhances the reliability of the findings. An important limitation of this study is the low patient number, which will be addressed in the future through a larger participant study. However, this is a known challenge with in vitro studies utilizing human tissue.

## Conclusions

In this study, we presented a comparative analysis of normal CPCs and patient-matched CPCs and MSCs, revealing that adult human articular cartilage contains a population of progenitor cells, termed cartilage chondroprogenitor cells, which exhibit MSC-like characteristics. These results support the concept that adult articular cartilage harbors a resident stem/progenitor cell population crucial for cartilage regeneration in OA treatment. Furthermore, this research highlights the feasibility of isolating patient-matched CPCs and MSCs, which is particularly promising for developing osteochondral tissues and offers potential applications in regenerative medicine and cartilage repair strategies.
